# Increase in environmental temperature affects exploratory behaviour, anxiety and social preference in *Danio rerio*

**DOI:** 10.1038/s41598-020-62331-1

**Published:** 2020-03-25

**Authors:** E. Angiulli, V. Pagliara, C. Cioni, F. Frabetti, F. Pizzetti, E. Alleva, M. Toni

**Affiliations:** 1 Department of Biology and Biotechnology “Charles Darwin”, Sapienza University, Rome, Italy; 2 Department of Experimental, Diagnostic and Specialty Medicine, University of Bologna, Bologna, Italy; 3 Center for Behavioural Sciences and Mental Health, Istituto Superiore di Sanità, Rome, Italy

**Keywords:** Social behaviour, Stress and resilience

## Abstract

The aim of this work is to investigate the effect of a temperature increase on the behaviour of adult zebrafish (*Danio rerio*) maintained for 21 days at 34 °C (treatment) and 26 °C (control). The temperatures chosen are within the vital range of zebrafish and correspond to temperatures that this species encounters in the natural environment. Previous results showed that the same treatment affects the brain proteome and the behaviour of adult zebrafish by producing alterations in the proteins involved in neurotransmitter release and synaptic function and impairing fish exploratory behaviour. In this study, we have investigated the performance of treated and control zebrafish during environmental exploration by using four behavioural tests (novel tank diving, light and dark preference, social preference and mirror biting) that are paradigms for assessing the state of anxiety, boldness, social preference and aggressive behaviour, respectively. The results showed that heat treatment reduces anxiety and increases the boldness of zebrafish, which spent more time in potentially dangerous areas of the tank such as the top and the uncovered bright area and at a distance from the social group, thus decreasing protection for the zebrafish. These data suggest that the increase in ambient temperature may compromise zebrafish survival rate in the natural environment.

## Introduction

Temperature is an abiotic parameter that is critical for animals, particularly for ectotherms whose body temperature depends on the environmental temperature. A vast literature from the 1800s shows the importance of temperature in the life of fish^1^. Temperature has been defined as the “abiotic master factor” for fish^2^ because it is one of the most important water parameters that affects fish biology, and for this reason, it must be considered in research and aquaculture to ensure animal welfare^3,4^. Variations in environmental temperature strongly affect fish biology not only by influencing growth, reproduction, spontaneous activity and metabolism^5^, as is well known for ectothermic animals, but also by affecting neurochemical parameters and behaviour^6–8^.

A common response of fish to temperature variation is the migration to habitats with more suitable temperatures^9,10^. However, if a fish cannot reach a habitat with optimal thermal conditions due to natural or anthropogenic factors^11,12^ or due to the loss of this habitat as a result of climate change, it will experience this temperature as stressful, which will activate compensatory mechanisms to restore homeostasis. Acute exposure (hours or days) to sub-optimal temperature results in a stress response, whereas longer-term exposure (weeks or months) results in an acclimatization response^10^.

Teleosts are widely used in ecological, behavioural and neurobehavioural studies since several lines of evidence have demonstrated that complex behaviours such as anxiety, aggression, learning and memory are conserved throughout the vertebrates^13,14^ and that behavioural indicators can be used to monitor environmental contamination^7^ and evaluate the impact of sublethal doses of pollutants^15^ on fish survival^16^. Therefore, given their ecological and economic importance, teleost fish are relevant models for studying the effects of short- and long-term thermal variation^17,18^.

Zebrafish (*Danio rerio*) are one of the species most used in research in fields ranging from developmental biology to neurobehaviour^19,20^ and ecotoxicology^21,22^. Zebrafish can also be conveniently used for studying the effects of thermal variation given its wide thermal tolerance (6.7 to 41.7 °C)^23,24^. Wild zebrafish inhabit slow-moving or standing water basins characterized by rapid daily (0.1–5.6 °C)^25^ and seasonal (6 °C in winter to above 38 °C in summer) variations in temperature^26^. This makes it possible to acclimate experimental animals to high or low temperatures in aquaria while still remaining in the range tolerated by the animal.

With the aim of investigating the impact of thermal variation on the nervous system and zebrafish behaviour, we have recently analysed the effects of temperature on the brain proteome of adult zebrafish exposed for 21 days to low (18 °C) and high (34 °C) temperatures and provided evidence that the thermal treatment up- or downregulates the expression of proteins involved in cytoskeletal organization, mitochondrial regulation, energy metabolism and synaptic functioning in the central nervous system (CNS)^8^. Behavioural tests performed in the same study using a Y-Maze apparatus also revealed significant alterations in zebrafish behaviour that could be related to the impairment of specific cognitive abilities caused by thermal treatment^8^.

Thus, the aim of the present study is to further investigate the effect of exposure to high temperature (34 °C) for 21 days on behaviours related to anxiety, boldness, aggression and social preference in zebrafish. Four experimental paradigms were used: the novel tank diving test (NTT), the light and dark preference test (LDT), the social preference test (SPT) and the mirror biting test (MBT). These tests, which are widely used in adult zebrafish, are based on the innate behaviours of the animal and allow the identification of behavioural variation following specific treatments. The NTT and LDT are based, respectively, on geotaxis and scototaxis^27^. The first test is the analogue of the open field test in rodents and is based on the innate escape “diving” behaviour of zebrafish in novel environments, whereas the second test is based on the innate tendency of adult zebrafish to prefer dark regions with respect to brightly lit areas. The SPT and NBT are used to assess the social behaviour phenotype in adult zebrafish^28^. The first test is based on the social nature of the zebrafish, which exhibits a strong social preference for swimming in a group when placed in a tank with conspecifics, whereas the second test is an experimental paradigm for assessing zebrafish boldness and aggressive behaviour. This test is based on the behavioural reaction that is stimulated by the image reflected in a mirror, to which the zebrafish can respond aggressively by biting it. The battery of four tests used in this study allows the evaluation of the impact of a thermal increase on the animal’s anxiety and boldness.

## Methods

### Ethical note

Animal experiments were performed in accordance with the guidelines approved by the Animal Care Committee and authorized by the Italian Ministry of Health (protocol number 290/2017-PR), and the animals were handled in accordance with the European directive 2010/63 on the protection of animals used for scientific purposes. The health status and the well-being of all animals involved in the study were checked daily for the duration of the thermal treatment and the subsequent behavioural tests. No procedures caused significant pain or lasting harm to the zebrafish, and no experimental subject died during the experimental procedures (fish housing, heat treatment and behavioural tests).

### Subjects

A total of 30 experimentally naïve adult (12 months old) zebrafish (50:50 male:female, mean mass 0.3 g) belonging to the same strain (AB) were obtained from a stock colony housed in the zebrafish facility of the University of Bologna. According to standard procedures, the zebrafish were housed at 26 ± 1 °C in a stand-alone rack system with transparent polycarbonate tanks and a dedicated water supply^29^. The zebrafish were raised and manipulated equally under a 14/10 hour light/dark photoperiod (light 6 am-8 pm).

The number of experimental subjects was chosen, given the reduction criteria, on the basis of data in the literature suggesting that in zebrafish, significant data may be obtained with n = 12–15 per group for strong effects^30^.

### Thermal treatment

Zebrafish were randomly transferred into two home tanks (15 individuals per tank, density of 1 zebrafish/l) and maintained at 26 °C (control temperature) for 10 days to acclimate them to the tank (adaptation period). The water temperature was then gradually raised in one tank from 26 °C to 34 °C over 72 hours. The treated zebrafish were then maintained at 34 ± 1 °C for 21 days (thermal treatment), and the control fish were maintained at 26 ± 1 °C.

### Fish husbandry during adaptation and thermal treatment

Zebrafish husbandry was based on the same protocol used previously^8^, and all procedures were conducted in the same laboratory room using the same home tanks and housing conditions previously described. Briefly, two identical glass home tanks (width (W) 40 x depth (D) 30 x height (H) 30 cm) were used. The interior enrichment of each tank (consisting of a heating coil, inlet and outlet pipes for the filters and an aerator) was replicated exactly. The water used throughout the experimental phase was produced by reverse osmosis pumps (Reverse Osmosis AquiliOS2) and maintained at the appropriate salinity by adding aquarium salt (1 g/l, Aqua Medic 301.01). In each tank, a constant flow of filtered water (600 l/h) was maintained by an external filtration system (Eden 511 h), and the water was also continuously aerated (7.20 mgO_2_/l) by an aquarium aerator (SicceAIRlight, 3300 cc/min 200 l/h). The water temperature was held constant by digital thermostats (Eden 430) connected to a heating coil (Eden 415, 230 V, 50/60 Hz, 80 W) and checked daily using an analogue thermometer.

The main chemical/physical characteristics of the tank water were checked at least two times per week using a Sera Aqua-test Box Kit (Sera, Italy) and an eSHa Aqua Quick Test (Nayeco, Spain). No differences were found between the treatment and control tanks. Total ammonia (NH_3_/NH_4_) and nitrite (NO_2_) were not detected (i.e., remained below the detection limit of 0.05 mg/l) and the water was partially renewed whenever the nitrate concentration reached 25 mg/l. The water pH was 7, the conductivity ranged between 400 and 500 micro-Siemens, the degrees of total hardness ranged between 7 and 14 and the degrees of carbonate hardness (dKH) was 6. The phosphate (PO_4_) levels were <1 mg/l, and no copper (Cu) or chlorine (Cl_2_) was detected.

**Figure 1:**
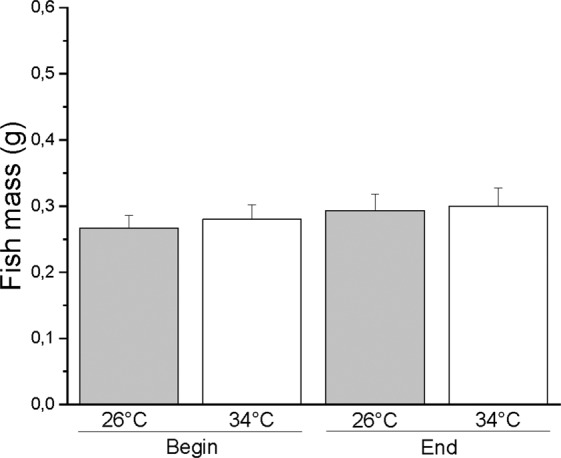
Zebrafish mass at the beginning and end of the thermal treatment. Data are expressed as the mean ± S.E.M. and were analysed by two-way ANOVA. No significant differences were detected (p > 0.33).

The zebrafish were fed three times a day (10 am, 2 pm and 6 pm) with a commercial dry granular food (TropiGranMIX, Dajanapet) by using automatic fish feeders (Eden 90, Eden Water paradise, Germany). Food (0.5 g/day) was administered to each tank, allowing zebrafish at both temperatures to feed themselves according to their appetite. The average mass (±SEM) at the beginning and at the end of the thermal treatment was, respectively, 0.27 ± 0.02 g and 0.29 ± 0.02 g at 26 °C and 0.28 ± 0.02 and 0.30 ± 0.03 at 34 °C. A two-way ANOVA (2×2, temperature x start/end treatment) analysis detected no statistically significant differences among masses (P > 0.33) (Supplementary Table S1 and Fig. 1). At the end of the behavioural tests, the average total length of the zebrafish was 3.18 ± 0.07 cm at 26 °C and 3.16 ± 0.06 cm at 34 °C, and a Student’s t-test for unpaired samples detected no difference in zebrafish length between the two temperatures (P > 0.83).

The salinity was checked with a handheld refractometer. Faeces and uneaten food were removed from the tanks at least three times per week. During the tank-cleaning operations, a water exchange of approximately 20–30% per week was performed to restore the correct volume of water and to maintain its chemical and physical parameters.

### Behavioural tests: general design

The day after the end of the thermal treatment (22^nd^ day), a total of 12 individuals from each home tank was subjected to a battery of four tests to evaluate different aspects of behaviour; the remaining 3 zebrafish were stored for future mRNA expression analysis. To minimize the interference between the different tests^31,32^, the NTT and LDT were performed alternately as the first or second test, whereas the SPT and MBT were performed alternately as the third or fourth test. Each individual zebrafish was captured by using a beaker and transferred from the home tank to the waiting tank (W15 x D10 x H10 cm) for 30 min until the beginning of the behavioural test^8^. The temperature of the water in all the tanks used in the behavioural tests was the same as the home tank in which the animal had been housed and was held constant at 26 °C or 34 °C with a maximum variation of 1 °C between the beginning and the end of each test. The duration of each test was 10 min. After each test, the zebrafish was transferred directly into the experimental tank for the next behavioural test using a beaker. After each test, the water was removed, and the apparatus was rinsed and filled with clean water. The tanks were not aerated during testing to avoid disturbing the animals. In the room, diffuse lighting was used to avoid directional lighting that could interfere with zebrafish behaviour. All tests were video-recorded by a webcam (Logitech C170) that was placed one metre above (LDT) or in front of (NTT, SPT and MBT) of each apparatus. The tests were conducted in May 2017 between 10 am and 5 pm.

### Novel tank diving test

**Figure 2:**
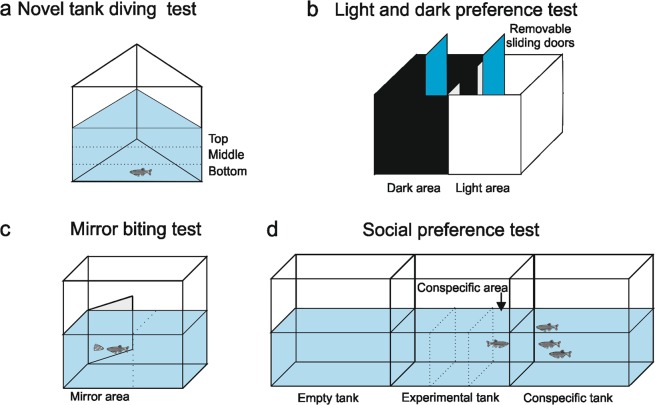
Schematic representation of the experimental design. The adult zebrafish were maintained for 21 days at 26 °C (control) and 34 °C (treatment). After thermal treatment, the fish were subjected to a battery of behavioural tests: NTT (**a**), LDT (**b**), MBT (**c**) and SPT (**d**). Behaviour was video-recorded for 10 min in each test.

Individual zebrafish were subjected to the NTT to evaluate locomotor activity and anxiety. The apparatus consisted of a glass transparent tank with a lower triangular base^33^ and 30-cm sides and was filled with 4.7 l of water (Fig. 2a). To measure vertical exploratory activity, the tank was virtually divided into three equal horizontal areas (bottom, middle and top). The parameters recorded were the number and duration of immobile phases (sec), distance travelled (m), average and maximum speed (m/sec), total number of rotations, ratio between clockwise (CW) and counter-clockwise (CCW) rotations, absolute turn angle (the sum of the absolute angles between each movement vector of the animal, in degrees), number of bottom/middle/top entries, distance travelled within the bottom/middle/top area (m), time spent in the bottom/middle/top area (sec), meandering (the change in direction of movement relative to the distance moved, whose value is the result of the absolute turn angle divided by the total distance travelled in deg/m) and latency to enter the top area (sec). In addition, the number of transitions to the top area, time spent and distance travelled in the top area were measured over time at 1-min intervals throughout the duration of the test.

### Light and dark preference test

The LDT was performed in a rectangular plastic tank (L33 × W18 × H18 cm) divided into two compartments of equal size (Fig. 2b). The lateral walls and the base of one half of the apparatus consisted of black plastic, whereas the other half consisted of opaque white plastic. The dark side was shielded from ambient light with an opaque black lid^34^, and the tank was filled with 4 l of water. In the middle of the tank, two transparent transverse septa restricted the passage between the two areas (6 cm) to prevent the zebrafish from freely swimming from one area to the other. Two transparent sliding doors (W18 × H18 cm) defined a central area (W7 × D18 × H18 cm) that was half black and half white where the zebrafish was housed before starting the test. The experimental subject was placed in the central area and left to settle; the two sliding doors were then lifted simultaneously to allow the zebrafish to move between the black and white areas for 10 min. The parameters analysed were the time spent in the bright area (sec) and the number of passages between the two areas.

### Mirror biting test

The MBT apparatus consisted of a barrel tank (W28 × D25 × H16 cm) filled with 4 l of water and equipped with a mirror (D16 × H14 cm) (Fig. 2c). The zebrafish was placed in the tank and left to settle, and the mirror was then tilted at an angle of 22.5° on the long side of the tank^31,35,36^. The position of the mirror (whether the reflected image was closer to the left or right side of the aquarium) was balanced between the tests. The parameters analysed were the time spent in the mirror area (sec), number of times crossing the lines denoting the mirror area, number of times that the fish bit the mirror and latency to the first mirror bite (sec).

### Social preference test

The experimental setting in this test consisted of three rectangular tanks (W28 × D25 × H16 cm) (*empty*, *experimental* and *conspecific*) aligned side by side in a horizontal line (Fig. 2d). Each tank was filled with 4 l of water.

The *experimental* tank was located in the middle and houses the experimental subject. One of the two adjacent tanks contained three zebrafish of the same size and age as the test subject and represented the social stimulus (*conspecific* tank); the other tank was left *empty*. The position of the group (on the left or right of the experimental tank) was balanced between tests.

At the beginning of the test, two black panels were positioned between the tanks to prevent the experimental subject from seeing the other tanks. The zebrafish was placed in the tank and left to settle, and the two panels were then gently removed, and the fish was allowed to swim freely for 10 min. The experimental tank was divided into three virtual areas. The area closest to the conspecific tank was designated as the “social area”, where the fish was assumed to prefer visual interaction with conspecifics. The parameters analysed were the number of immobile episodes, time immobile (sec), total distance travelled (m), average and maximum speed (m/sec), total number of rotations, number of CW and CCW rotations, absolute turn angle (deg), meandering (deg/m), time spent in the social area (sec), distance travelled in the social area (m), and latency to first entry into the social area (sec).

### Video tracking and statistical analysis

**Figure 3:**
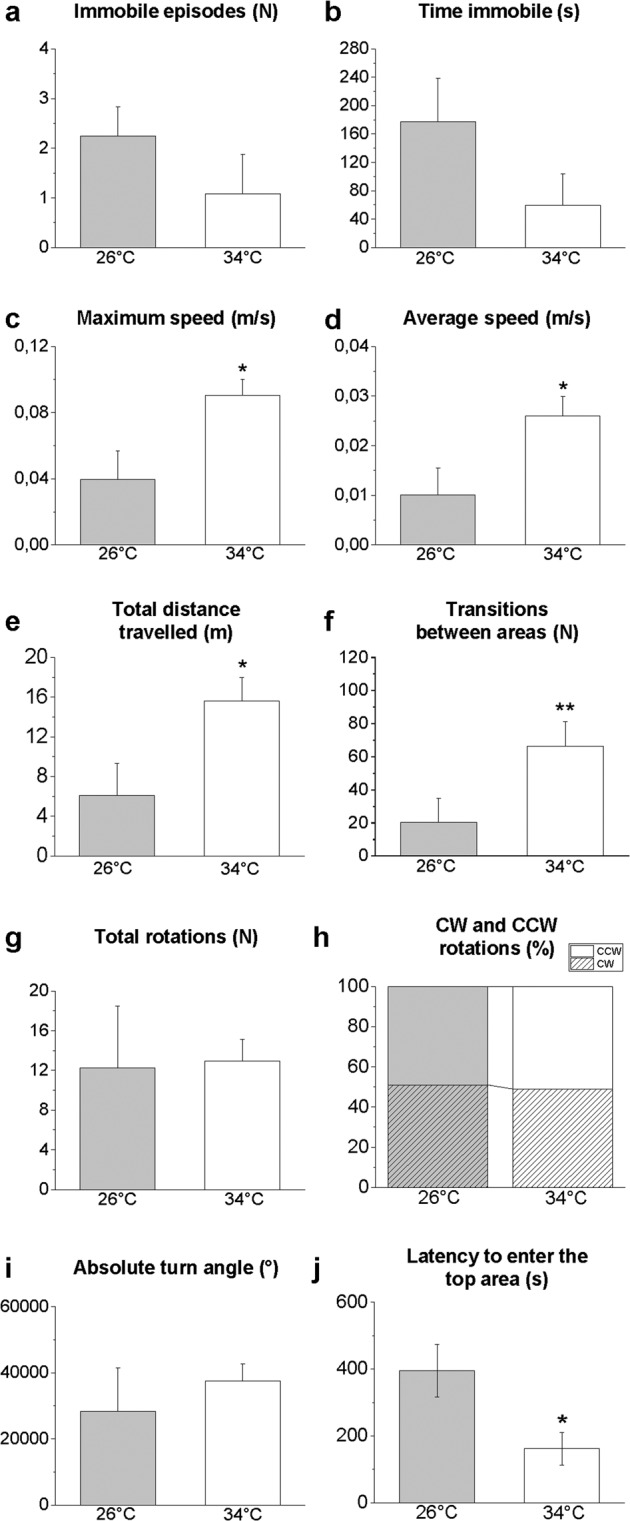
Novel environment exploration behaviour in the NTT. Immobile episodes (**a**), time immobile (**b**), maximum speed (**c**), average speed (**d**), total distance travelled (**e**), transition between areas (**f**), total rotations (**g**), clockwise (CW) and counter-clockwise (CCW) rotations (**h**), absolute turn angle (**i**), and latency to enter the top area (**j**). Data are expressed as the mean ± S.E.M. and were analysed by unpaired Student’s t-test. *P ≤ 0.05; **P ≤ 0.01, N = 12.

**Figure 4:**
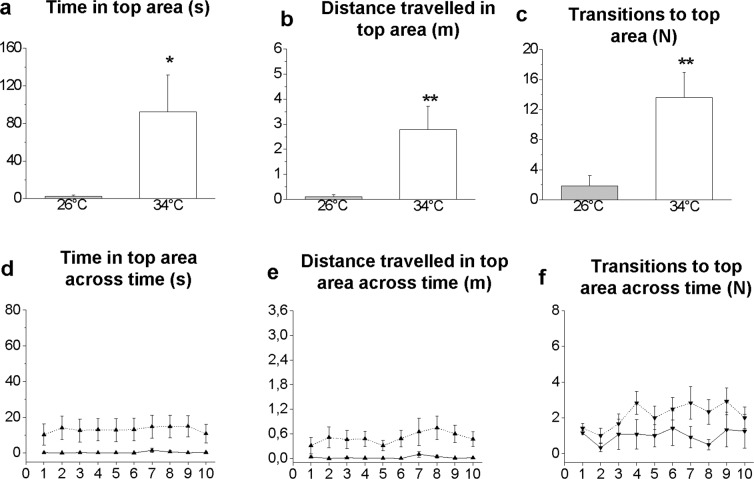
Top area exploration in the NTT. Time spent (**a**,**d**), distance travelled (**b**,**e**) and number of transitions (**c**,**f**). Total (**a–c**) and 1-min interval (**d–f**) analyses are reported. Data are expressed as the mean ± S.E.M. and were analysed by unpaired Student’s t-test (**a–c**) and by repeated measures ANOVA (**d–f**). *P ≤ 0.05; **P ≤ 0.01, N = 12. (**d–f**) Solid and dotted lines refer to 26 °C and 34 °C, respectively.

**Figure 5:**
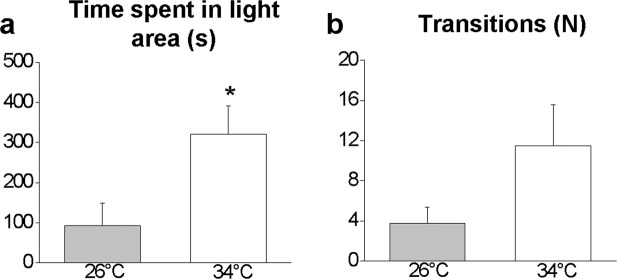
Scototaxis behaviour in the LDT. Time spent in the bright area (**a**) and number of passages between bright and dark areas (**b**). Data are expressed as the mean ± S.E.M. and were analysed by unpaired Student’s t-test. *P ≤ 0.05; N = 12.

**Figure 6:**
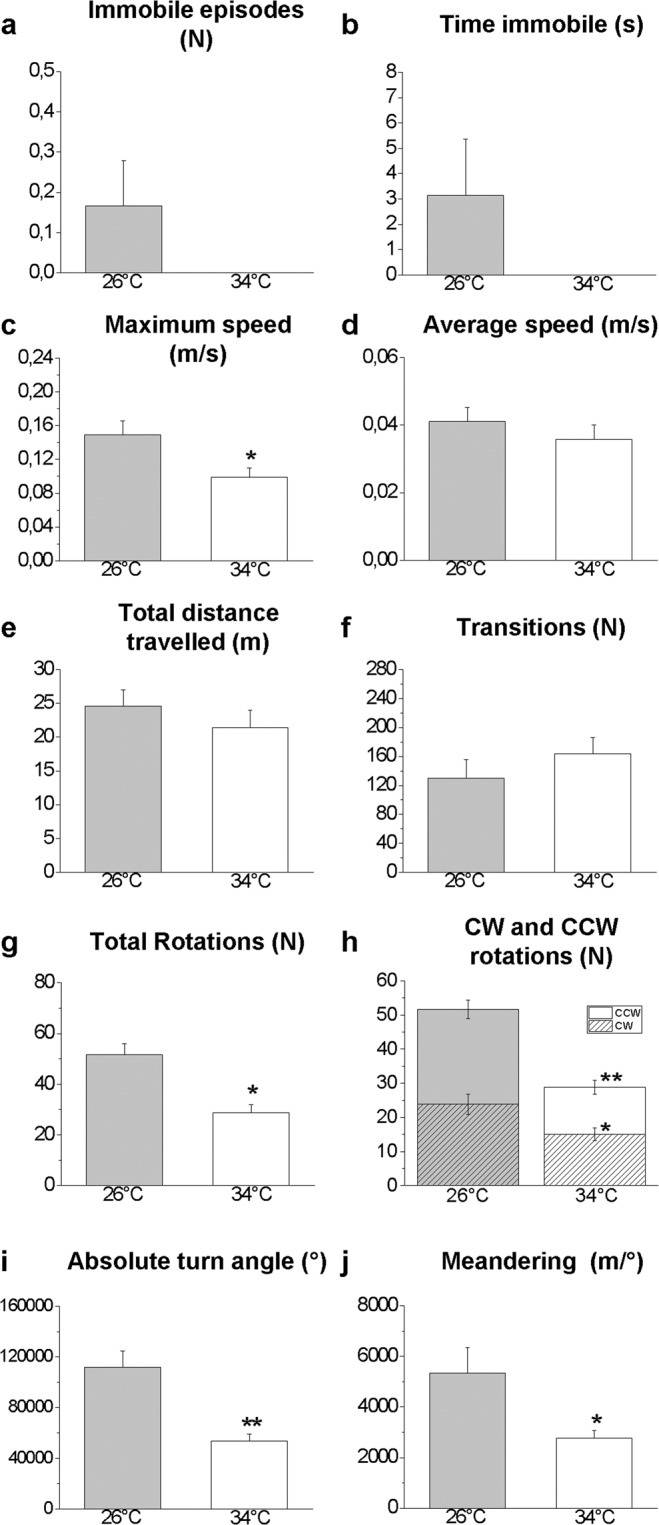
Shoaling behaviour in the SPT: whole tank analysis. Immobile episodes (**a**), time immobile (**b**), maximum speed (**c**), average speed (**d**), total distance travelled (**e**), transition between areas (**f**), total rotations (**g**), clockwise (CW) and counter-clockwise (CCW) rotations (**h**), absolute turn angle (**i**), and meandering (**j**). Data are expressed as the mean ± S.E.M. and were analysed by unpaired Student’s t-test. *P ≤ 0.05 **P ≤ 0.01, N = 12.

**Figure 7:**
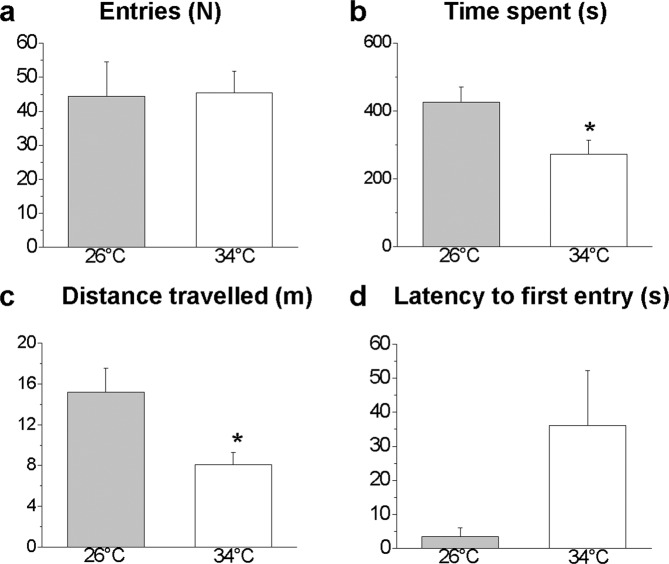
Shoaling behaviour in the SPT: conspecific area analysis. Parameters analysed in the conspecific compartment: number of entries (**a**), time spent (**b**), distance travelled (**c**), and latency to first entry (**d**). Data are expressed as the mean ± S.E.M. and were analysed by unpaired Student’s t-test. *P ≤ 0.05, N = 12.

**Figure 8:**
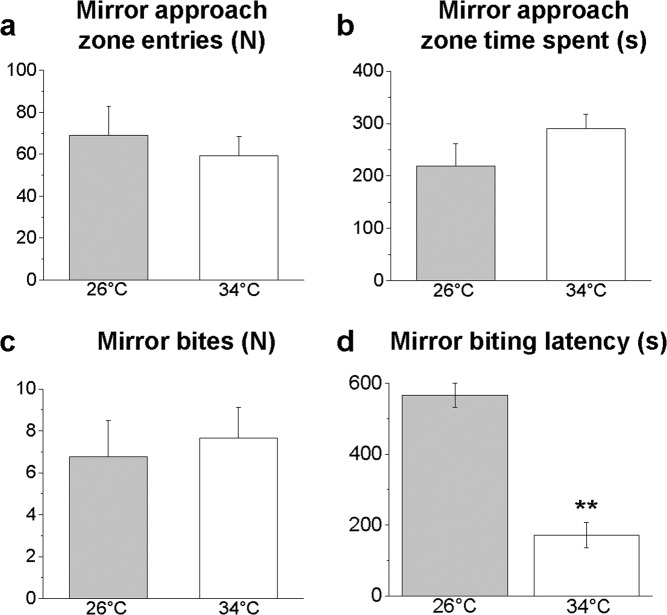
Aggressive behaviour in the MBT. Mirror approach area: entries (**a**), time (**b**), mirror bites (**c**) and mirror-biting latency (**d**). Data are expressed as the mean ± S.E.M. and were analysed by unpaired Student’s t-test; **P ≤ 0.01, N = 12.

The recording sessions lasted 10 min each and videos were analysed using the ANY-Maze® software (trial version, Stoelting Co., Wood Dale, IL, USA). The results were expressed as the mean ± SEM. The data were subjected to a Student’s t-test for unpaired samples (data shown in Figs. 3–8) or an ANOVA with a post hoc test utilizing a Bonferroni correction (data shown in Figs. 1 and 4d–f). Differences were considered to be statistically significant at *P* ≤ 0.05. All statistical analyses were performed using Origin Pro 2018 software. CorelDRAW® X7 and Microsoft Power Point were used for figure design.

## Results

### Novel tank diving test

The NTT evaluates the response to anxiety evoked by novelty. Although not statistically significant, a reduction in the number (P = 0.2473, Fig. 3a) and duration (P = 0.1304, Fig. 3b) of immobile episodes (freezing) was observed in treated zebrafish. The parameters related to animal locomotor and swimming activity such as the maximum (P = 0.0181, Fig. 3c) and average (P = 0.0270, Fig. 3d) speed, total distance travelled (P = 0.0266, Fig. 3e), and the number of line crossings between the horizontal areas (bottom, middle and top) (P = 0.0414, Fig. 3f) significantly increased in zebrafish maintained at 34 °C compared with the controls. No significant differences (P > 0.05) were found in the total number of rotations (Fig. 3g), the ratio of CW and CCW rotations (Fig. 3h) or the absolute turn angle (Fig. 3i). Moreover, at 34 °C, a lower latency (P = 0.0192) to enter the top area was observed compared with the control subjects (Fig. 3j).

Vertical exploration increased in zebrafish at 34 °C compared with zebrafish at 26 °C, as indicated by a longer time spent (P = 0.0305, Fig. 4a), distance travelled (P = 0.0090, Fig. 4b) and number of transitions by fish (P = 0.0038, Fig. 4c) in the top area. To assess whether exploration of the top area was modulated over time, a more detailed analysis was performed at 1-min intervals using a two-way repeated measures ANOVA (2×10, temperature x time). No differences in exploratory activity over time were observed as measured by the time spent in the top area (F_9,198_ = 0.8965, P = 0.5294, Fig. 4d), distance travelled in the top area (F_9,198_ = 1.0264, P = 0.3731, Fig. 4e) or transitions to the top area (F_9,198_ = 1.2079, P = 0.2919, Fig. 4f).

### Light and dark preference test

The analysis of the results from the LDT showed that the adaptation to 34 °C involves an increased preference for the bright area, where treated zebrafish spent more time (53.5% of the total time) than the control zebrafish (15.5% of the total time) (P = 0.0197, Fig. 5a). Treated zebrafish also exhibited a tendency for a higher number of crossings between the two compartments of the apparatus (P = 0.0888, Fig. 5b).

### Social preference test

In the SPT, no significant differences were found in the number (P = 0.1522, Fig. 6a) or duration (P = 0.1728, Fig. 6b) of immobility events or in the average speed (Fig. 6d) or distance travelled (Fig. 6e) between treated and control subjects. Zebrafish at 34 °C showed a significant reduction in the maximum speed (P = 0.0193, Fig. 6c), total number of rotations (P < 0.0004, Fig. 6g), CW (P = 0.0206) and CCW (P < 0.0004) (Fig. 6h) rotations, absolute turn angle (P < 0.0004, Fig. 6i) and meandering (P = 0.0211, Fig. 6j).

Although the number of entries into the conspecific area was similar in treated and control zebrafish (P = 0.9282, Fig. 7a), a significant decrease in the time spent (P = 0.0190, Fig. 7b) and the distance travelled (P = 0.0126, Fig. 7c) was measured at 34 °C. Treated fish exhibited a tendency for increased latency to first entry into the social area (P = 0.0566, Fig. 7d).

### Mirror biting test

In the MBT, no statistically significant differences were found in the number of entries to (P = 0.5682, Fig. 8a) or in the time spent in (P = 0.1722, Fig. 8b) the mirror approach zone, or in the number of bites (P = 0.6908, Fig. 8c), whereas a significant reduction in the latency to the first bite was observed in the zebrafish at 34 °C (P < 0.0001, Fig. 8d).

## Discussion

In the present study, we documented the effect of a temperature increase on adult zebrafish by providing evidence that exposure to 34 °C for 21 days changes behavioural parameters compared with the control temperature (26 °C). The experimental subjects underwent four behavioural tests that allowed us to evaluate different aspects of behaviour such as vertical exploration of the water column (NTT), scototaxis (LDT), social interest (SPT) and aggression (MBT). The results indicate that heat treatment alters the animal’s anxiety state and the behaviour of zebrafish in a new environment.

In the NTT, anxiety-like behaviour consists of dwelling on the bottom of the tank, whereas exploration of the more superficial zones corresponds to a greater boldness of the animal^34,37–39^. This has been confirmed by experiments conducted on zebrafish using the NTT, in which the administration of anxiolytic compounds such as desipramine, citalopram, diazepam and LSD^33,38,40^ increased the exploration of the surface regions. Consistent with published data, in our experiments, the control fish dwelt mainly at the bottom and exhibited freezing events^37,39–41^, whereas the heat-treated fish spent more time in the top area, suggesting an anxiolytic effect.

To further investigate how the thermal increase alters the natural behaviour of the animal, zebrafish were subjected to a LDT that assesses an animal’s preference for staying in dark protected environments or exploring bright open environments, where the risk of being identified and predated is higher. In this test, an increase in the time spent in the bright compartment following a specific treatment is interpreted as an anti-anxiety effect^42^, and it has been reported that treatment with anxiolytic compounds such as chlordiazepoxide and ethanol increase the preference for bright areas in zebrafish^33^. In our study, zebrafish maintained at 34 °C increased the number of passages between dark and light sectors as well as the time spent in the bright area, which shows an increased motivation for exploring the open environment despite the possible risk. In our study, the dark compartment was created with black walls and by covering the dark sector with cardboard, as has been done previously^31,34,43^. A recent study has shown that, depending on the type of apparatus and the experimental protocol used, the LDT can confuse the preference for a shaded background with a preference for illumination level^44^. According to these observations, the dark area used in our study, which was obtained by covering one entire side, constitutes a cave-like area that in the natural environment could correspond to shelters behind stones and leaves, and the light area corresponds to an open environment such as the middle of a pond far from the grass and the foliage.

To assess whether heat treatment influences social behaviour and the interest in conspecifics, we conducted experiments using the SPT. Zebrafish at 26 °C showed interest in conspecifics and this reflects the social and cooperative nature of the animal^45^. Numerous studies agree that the main function of shoaling is predator avoidance and that an increase in shoaling can be associated with increased anxiety^46–50^. In support of this interpretation, it has been reported that the use of anxiolytic drugs reduces shoaling in zebrafish^50,51^. In our study, heat treatment reduced the interest in conspecifics. This behaviour suggests an anxiolytic effect of thermal increase that could be caused by a reduced ability of the experimental subjects to recognize conspecifics, a reduced interest in conspecifics or an actual reduction in anxiety.

Finally, the effect of heat treatment on the aggressiveness of zebrafish was tested using the MBT. At 34 °C, a shorter latency to the first bite was observed compared with the controls. Similarly, when the Neotropical cichlid fish *Cichlasoma paranaense* was subjected to a thermal increment from 27 °C to 33 °C, it showed an increased tendency for aggressive episodes, and significant differences were detected over a wider thermal range (21–33 °C)^52^. Overall, our MBT results are consistent with an increase in zebrafish boldness at high temperature.

Data collected in the present study demonstrate that adaptation for 21 days at 34 °C caused an impairment of natural zebrafish behaviour and corroborates previous results that showed how the same heat treatment can alter the expression of brain proteins that are involved in important cellular events such as synaptic plasticity and neurotransmitter release, thereby altering zebrafish cognitive abilities^8^. This suggests that the behavioural alteration induced by heat treatment may be a consequence of neurochemical alterations. However, given the complexity of the alterations induced by temperature in the CNS, any direct association between the variation in a single protein or a single pathway and changes in behaviour is purely speculative, and further studies are needed to circumscribe the neurochemical alterations underlying the observed behavioural changes.

The results of the four behavioural tests show that zebrafish at 34 °C reduce the time spent in safe environments as represented by the bottom of the tank, the dark/protected area and shoal cohesion and instead perform forays into potentially risky environments such as the top area of the tank, the uncovered/bright area and zones far from the social group that could guarantee better protection. The alteration of the CNS revealed by previous proteomic studies thus appears to alter the exploratory behaviour of zebrafish.

Although the purpose of the tests performed was not to mimic natural conditions, it is nevertheless interesting to note that the behaviours shown by experimental subjects at 34 °C would expose zebrafish living in the natural environment to a greater risk of predation. Given that the zebrafish lives in regions of South-East Asia that are characterized by wide seasonal variations ranging from 6 °C in winter to more than 34 °C in summer^26^, and that predictive scenarios estimate a temperature increase of 1.4–5.8 °C by 2100^53,54^, it can be assumed that in the future, zebrafish will be exposed more frequently to a temperature of 34 °C in the natural environment. Future experiments will be conducted by our group to verify whether the same effects observed in this study after 21 days can be induced by shorter exposure (a few days) at the same temperature. These studies will contribute to an understanding of the impact of temperature increases on zebrafish behaviour.

## Supplementary information

Supplementary Table S1.

## Data Availability

The datasets generated during the current study are available in the *figshare* repository (10.6084/m9.figshare.9861218).
