# Supplementary material for: Increase in environmental temperature affects exploratory behaviour, anxiety and social preference in Danio rerio
Source: Sci Rep. 2020 Mar 25;10:5385. doi: 10.1038/s41598-020-62331-1 (PMC7096496; doi:10.1038/s41598-020-62331-1)
Supplement: Supplementary file 1 — Supplementary Table S1. [file 41598_2020_62331_MOESM1_ESM.docx]

**SUPPLEMENTARY INFORMATION for**

**Increase in environmental temperature affects exploratory behaviour, anxiety and social preference in *Danio rerio*.**

**E. Angiulli^1^, V. Pagliara^1^, C. Cioni^1^, F. Frabetti^2^, F. Pizzetti^2^, E. Alleva^3^, M. Toni^1*^**

^1^Department of Biology and Biotechnology ‘‘Charles Darwin”, Sapienza University, Rome, Italy

^2^Department of Experimental, Diagnostic and Specialty Medicine, University of Bologna, Italy

^3^ Center for Behavioural Sciences and Mental Health, Istituto Superiore di Sanità, Rome, Italy

**Current file content:**

- **Supplementary Table S1**

**Supplementary Table S1**

Mass (g) of fish at the beginning and end of the 21-day thermal treatment.

| **Beginning** | |  | **End** | |
| --- | --- | --- | --- | --- |
| **26 °C** | **34 °C** |  | **26 °C** | **34 °C** |
| 0.2 | 0.2 |  | 0.5 | 0.6 |
| 0.2 | 0.2 |  | 0.4 | 0.4 |
| 0.2 | 0.2 |  | 0.4 | 0.3 |
| 0.2 | 0.2 |  | 0.3 | 0.4 |
| 0.2 | 0.2 |  | 0.3 | 0.3 |
| 0.2 | 0.2 |  | 0.4 | 0.3 |
| 0.2 | 0.3 |  | 0.3 | 0.2 |
| 0.3 | 0.3 |  | 0.3 | 0.3 |
| 0.3 | 0.3 |  | 0.2 | 0.2 |
| 0.3 | 0.3 |  | 0.2 | 0.2 |
| 0.3 | 0.3 |  | 0.2 | 0.3 |
| 0.3 | 0.3 |  | 0.3 | 0.3 |
| 0.3 | 0.3 |  | 0.2 | 0.2 |
| 0.4 | 0.4 |  | 0.2 | 0.3 |
| 0.4 | 0.5 |  | 0.2 | 0.2 |
